# A Mindfulness Application for Reducing Prenatal Stress

**DOI:** 10.1111/jmwh.13359

**Published:** 2022-04-11

**Authors:** Anne C. Porter, Sharon Hunter, Kate Noonan, M. Camille Hoffman

**Affiliations:** ^1^ Department of Obstetrics and Gynecology University of Colorado School of Medicine Aurora Colorado; ^2^ Department of Psychiatry University of Colorado School of Medicine Aurora Colorado

**Keywords:** prenatal stress, prenatal depression, mindfulness, applications

## Abstract

**Introduction:**

Up to 40% of patients report depression or anxiety symptoms in pregnancy; feelings of increased stress are nearly universal. Antepartum stress is linked to adverse outcomes including preterm birth, low birthweight, postpartum depression, and maternal self harm. Unfortunately, limited treatment options exist, and patients are often hesitant to initiate medications prenatally. Thus, the development of efficacious nonpharmacologic interventions is crucial. This pilot study investigated the feasibility and impact of an application (app)‐based mindfulness practice, begun in the first trimester, on maternal stress and pregnancy outcomes.

**Methods:**

The study enrolled patients prior to 15 weeks’ gestation and followed them prospectively through birth. Patients were provided with a free subscription to Expectful, a commercially available prenatal mindfulness app, and asked to complete daily meditations. Patients completed the Perceived Stress Scale (PSS) self‐assessment at 15 weeks and 28 weeks. PSS scores and pregnancy outcomes were compared with a historical control group of pregnant people who did not use the app.

**Results:**

Of 68 patients approached, 59 consented to enrollment. Of these, 21 used the app, with an average use of 170 minutes (range, 1.3‐1315 min). The average PSS score was significantly lower in the app group at 28 weeks. Additionally, the change in PSS score for app users was greater compared with that of the historical control between enrollment and 28 weeks (−6.3 vs −0.95*, P* = .0008). Pregnancy outcomes were similar for app users and the historical control.

**Discussion:**

Our recruitment rate suggests pregnant patients are eager for a nonmedication intervention to decrease stress. However, adherence after enrollment was limited. For a subset of motivated patients, an app‐based mindfulness practice significantly reduced perceived stress between the second and third trimesters compared with non‐app users. Prenatal mindfulness apps represent an important low‐intervention, low‐cost, highly accessible tool for managing perinatal mood and stress.

## INTRODUCTION

Antepartum maternal stress, even in the absence of diagnoses of anxiety and depression, is associated with a wide range of adverse pregnancy outcomes for both the woman and the child. Prenatal stress is linked to increased risk of preterm birth^1^, ^2^, ^3^, ^4^, ^5^, ^6^, ^7^ and low birth weight.^3^, ^5^, ^7^, ^8^, ^9^ After birth, antepartum stress increases the risk of postpartum depression^10^, ^11^ and impacts maternal‐neonatal bonding.^11^ Recent research suggests that prenatal stress has long‐lasting effects on childhood neurodevelopment.^12^, ^13^, ^14^ Therefore, it is critical that pregnant patients are given tools to decrease their stress levels to secure the best possible outcome for themselves and their children.

Treatment options for stress during pregnancy are limited. Pregnant patients and their health care providers must constantly weigh the benefits and potential risks of any given medication for the patient and the fetus.^15^ Moreover, patients are resistant to initiate pharmacologic treatment options for stress, depression, or anxiety during pregnancy.^16^ There are several nonpharmacologic treatment options for depression and stress during pregnancy, including interpersonal therapy, cognitive behavioral therapy, bright light therapy, massage therapy, and acupuncture. Although psychotherapy has clear benefits for patients experiencing antenatal depression, the findings regarding the efficacy of light therapy, massage therapy, and acupuncture have been mixed.^17^ Additionally, these therapies can be costly or inaccessible to many patients.^18^ Therefore, there is an urgent need for a nonpharmacologic, easily accessible option to help patients manage stress during pregnancy.

Mindfulness, defined as attention and awareness of the present moment without judgment,^19^ is widely used outside of pregnancy as a primary or adjunct treatment for stress, anxiety, and depression. A mindfulness practice has been shown to decrease several physiologic markers of stress including blood cortisol levels, C‐reactive protein levels, blood pressure, and heart rate.^20^ Within pregnancy, emerging evidence indicates that mindfulness improves depressive, anxiety, and stress scores. Furthermore, these studies show that mindfulness strategies lead to fewer relapses of postpartum depression, fewer sleep disturbances, and overall milder mood symptoms.^21^, ^22^ Intriguingly, a telehealth intervention that delivered mindfulness‐based cognitive behavioral therapy to groups of antepartum patients appeared to reduce symptoms of prenatal depression.^23^ This study suggests that patients can benefit from mindfulness outside of a traditional one‐on‐one, in‐person psychotherapy setting. Similarly, a recent online adaptation of mindfulness interventions for pregnancy has shown promising results in reducing depressive symptoms.^24^ Helping patients develop a mindfulness practice is an intriguing tool for combating antepartum stress.

**QUICK POINTS**

✦ Mindfulness applications (apps) are a feasible approach to increase access to mental health resources for pregnant patients.
✦ Establishing an app‐based mindfulness practice may reduce perceived stress in pregnant patients.
✦ Pregnant patients are eager for a nonpharmacologic intervention for stress reduction.

Although a mindfulness practice has been shown to improve outcomes for pregnant patients and their chidren,^21^, ^22^ the effectiveness and feasibility of a mindfulness smartphone application (app) on reducing stress and improving pregnancy outcomes has not yet been fully investigated. Emerging studies have shown promise in app‐based mindfulness practices for pregnant patients, with high feasibility and acceptability of these interventions.^25^ Excitingly, a recent randomized control trial demonstrated a significant improvement in depressive symptoms and anxiety outcomes for smartphone‐based mindfulness training in pregnancy.^26^ Apps have the benefit of being less costly than one‐on‐one mindfulness interventions and accessible to patients who live in regions with fewer mental health professionals.^18^

Expectful is a commercially available app designed to engage pregnant patients in a mindfulness practice. Expectful has an extensive library of guided imagery meditations and body scan exercises that are categorized by trimester and emotional state. These exercises were created with the help of licensed psychologists, hypnotherapists, and meditation experts. Our objective in this pilot study was to assess the feasibility and impact of an app‐based mindfulness practice, begun in the first trimester, on maternal stress and pregnancy outcomes.

## METHODS

### Study Participants

This prospective cohort study recruited healthy pregnant patients during routine prenatal visits for initial ultrasound before 15 weeks’ gestation at University of Colorado Obstetrics and Gynecology clinic sites from 2017 to 2020. A small team of individual providers recruited eligible patients with criteria as outlined. The study was not publicly advertised to patients for enrollment. Gestational age was assessed based on best clinical estimate using reported last menstrual period and first dating ultrasound. Patients were between 18 and 45 years of age, demonstrated English literacy, and had daily access to an iPhone or computer. Stress level prior to enrollment was not evaluated, as patients were included regardless of preexisting stress. Patients were excluded if they had multiple gestation, medical conditions requiring chronic corticosteroid use (topical, inhaled/oral), or a complex psychiatric history requiring higher‐level psychiatric care. Retention, app usage, and completion of study procedures were documented. Patient information, including demographic information and pregnancy outcomes, was retrieved from patient records. The project was approved by the Colorado Multiple Institutional Review Board, and all participants provided informed consent.

A previously established historical control group was used for comparison with app users in the prospective cohort. These patients were also recruited at prenatal visits during the first trimester or before 16 weeks’ gestation. Patients were between 18 and 45 years of age and planned to give birth at the Denver Health and Hospital Authority in Denver, Colorado. Inclusion and exclusion criteria for this cohort were similar to the current study.^1^

### Self‐Report Measures

Patients completed the Perceived Stress Scale (PSS),^27^ a self‐assessment questionnaire designed to evaluate how unmanageable, stressful, and overburdened an individual perceives their life over the previous month. The PSS was developed for those with at least a junior high school education. The survey consists of 14 questions with 7 positive items and 7 negative items that assess general rather than context‐dependent stress. For negative questions, scores are rated on a 5‐point Likert scale (0 = never; 1 = almost never; 2 = sometimes; 3 = fairly often; 4 = very often); for positive items, the scale is reversed. Scores range from 0 to 56; a higher score indicates a higher frequency of stressors, irritations, poor coping, anger, and difficulty in the preceding 4 weeks.^1^, ^27^ This self‐assessment scale has established internal consistency, confirmed test‐retest reliability, and proven factorial validity.^27^ In pregnancy, higher PSS values have been shown to be associated with preterm birth and lower birth weight.^5^, ^6^, ^28^

Patients from both the prospective cohort and the historical control group completed the PSS at approximately 15 and 28 weeks’ gestation. For patients in the prospective cohort, the PSS score at 15 weeks was collected prior to app usage, and the PSS score at 28 weeks was collected after app usage.

At enrollment, patients also completed the Adverse Childhood Experience (ACE) screen, a self‐assessment of a person's exposure to trauma during their childhood. The ACE screen defines traumas as those related to child abuse and exposure to household dysfunction, with scores ranging from 0 (no exposure) to 10. Child abuse includes psychological, physical, and sexual abuse; household dysfunction is defined as exposure to substance abuse, mental illness, violent treatment of a parent, or criminal behavior in the household.^29^

### App Use

Access to the Expectful mindfulness app was provided free of charge to all study participants. Meditations are tailored to trimester as well as particular physical and emotional states. Each meditation lasts 10 to 20 minutes. Daily app usage was recommended to those enrolled in the prospective cohort. The number of meditations started and total minutes of meditation performed were tracked by the Expectful app development team.

### Statistical Analysis

Not all patients completed all parts of the follow‐up assessments, which resulted in different sample sizes for different follow‐up analyses. ANOVA and Fisher's exact test were used to compare groups on demographic and study characteristics including gravidity and parity, ACE score, PSS score, psychiatric diagnosis, psychiatric medication use, gestational age at birth, and birthweight. ANOVA and *t* tests were used for mean comparison. Paired *t* test was used to compare 15‐week and 28‐week assessments within each cohort. All statistical analyses were performed on GraphPad Prism 7.0c (San Diego, California). Differences were considered significant when *P* < .05.

## RESULTS

### Participant Characteristics

**Figure 1:**
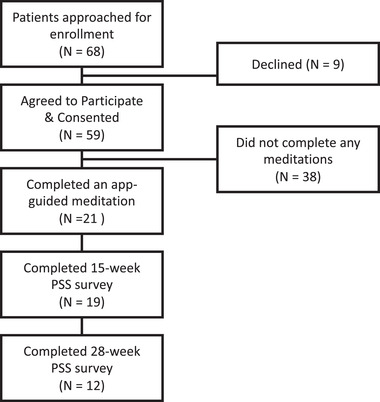
Study Enrollment and Participation Diagram Abbreviation: PSS, Perceived Stress Scale.

Figure 1 displays enrollment and participation numbers. From 2017 to 2020, a total of 68 patients were approached for enrollment, of whom 59 (84%) consented. Of the 59 patients who consented, 21 (36%) completed at least one meditation; these 21 participants were designated as app users. App users completed an average of 19 meditations with an average time of 170 minutes (range, 1.3‐1315 min). Among app users, 19 (90%) completed the 15‐week PSS assessment and 12 (57%) completed the 28‐week assessment. Multiple survey completion reminders were sent; reasons for lack of adherence were not assessed. No non‐app users completed the 28‐week PSS assessment, and thus, they were not included in analysis.

Demographics for all the patients who enrolled in the study are presented in Table 1. Of those who enrolled, app users had lower gravidity, parity, and prepregnancy body mass index but were otherwise similar. ACE scores were similar between app and non‐app users. Table 2 summarizes the demographic information for those in the prospective cohort who were app users and the historical control cohort of 247 patients. The historical control cohort was younger with higher mean gravidity and parity. There were no differences between groups in psychiatric history or psychiatric medication use.

**Table 1:** Participant Demographics of All Enrolled Participants in Prospective Cohort, Comparing App and Non‐App Users (N = 59)

Participant Characteristic	App (n = 21)	Non‐App (n = 38)	*P* Value
**Maternal age, mean (SD), y**	32.8 (4.7)	31.9 (4.5)	.46
**Gravidity, mean (SD)**	1.7 (0.83)	2.6 (1.7)	.04*
**Parity, mean (SD)**	0.3 (0.5)	0.95 (0.9)	**.007**
**Prepregnancy body mass index, mean (SD), kg/m^2^**	25.8 (5.2)	30.2 (6.8)	**.01**
**ACE score, mean (SD)**	1.8 (1.7)	1.5 (2.3)	.62
**PSS at 15 wk, mean (SD)**	23 (8.5)	18.9 (8.5)	.2
**Psychiatric history, n (%)**			.41
Yes	9 (43)	12 (32)	
No	12 (57)	26 (68)	
**Psychiatric medication use, n (%)**			>.999
Yes	3 (14)	7 (18)	
No	18 (86)	31 (82)	

Abbreviations: ACE, Adverse Childhood Experience; app, application; PSS, Perceived Stress Scale.

*Significant values are in bold.

**Table 2:** Participant Demographics of App Users Compared with a Historical Control Cohort Who Did Not Have Access to the App

Participant Characteristic	App (n = 21)	Control (n = 247)	*P* Value
**Maternal age, mean (SD), y**	32.8 (4.7)	29.1 (5.9)	.006*
**Gravidity, mean (SD)**	1.7 (0.83)	3.0 (1.8)	**.003**
**Parity, mean (SD)**	0.3 (0.5)	1.3 (1.3)	**.001**
**ACE score, mean (SD)**	1.8 (1.7)	2.6 (2.4)	**>.999**
**Prepregnancy body mass index, mean (SD), kg/m^2^**	25.8 (5.2)	27.7 (6.6)	.22
**Psychiatric history, n (%)**			.05
Yes	9 (43)	158 (66)	
No	12 (57)	81 (34)	
**Psychiatric medication use, n (%)**			.43
Yes	3 (14)	63 (25)	
No	18 (86)	188 (75)	

Abbreviations: ACE, Adverse Childhood Experience; app, application.

*Significant values are in bold.

### App Usage and PSS Change

**Figure 2:**
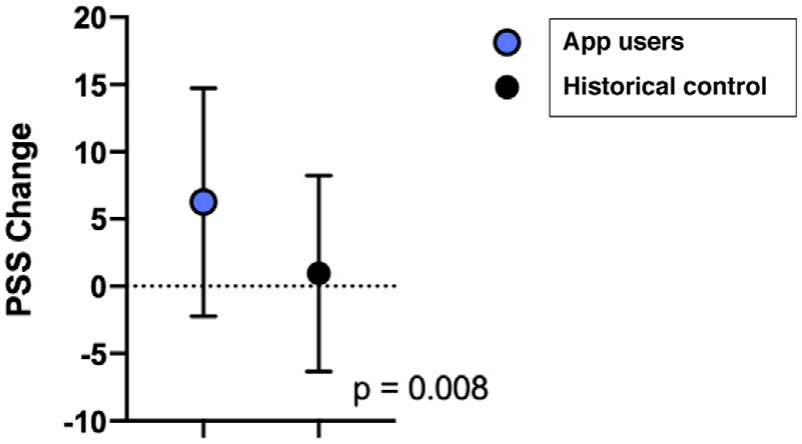
PSS Score Trajectories for App Users and Non‐App Users Abbreviation: PSS, Perceived Stress Scale.

Table 3 summarizes PSS scores across time for both the app users and the historical control. At the initial 15‐week assessment, app users and the historical control had similar PSS scores with a mean (SD) of 23.0 (8.5) and 23.02 (8.0), respectively. At the 28‐week assessment, app users with any duration of app use had significantly lower PSS scores (mean, 17.9; SD, 8.3) than the historical control (mean, 22.3;SD, 7.7). Furthermore, the change in PSS score for app users (mean, 6.3; SD, 8.5) was significantly greater compared with that of the historical control (mean, 0.95; SD, 7.3) (Figure 2). Critically, app users had a significantly lower PSS score at 28 weeks than at 15 weeks, whereas the PSS scores of the historical control were not significantly different between 15 weeks and 28 weeks. Together, these results suggest that an app‐based mindfulness practice may decrease perceived stress levels in pregnant patients.

**Table 3:** Perceived Stress Scores, Gestational Age at Birth, and Birthweight for App Users Compared with Historical Cohort

Outcomes	App Users (n = 19)	Control (n = 247)	*P* Value
PSS (15 wk), mean (SD)	23.0 (8.5)	23.02 (8.0)^a^	>.999
PSS (28 wk), mean (SD)	17.9 (8.3)^b^	22.3 (7.7)^c^	.03*
PSS change, mean (SD)	6.3 (8.5)^b^	0.95 (7.3)^c^	**.008**
Gestational age, mean (SD), wk	39.5 (0.9)^d^	39.1 (1.8)^e^	.39
Birthweight, mean (SD), g	3163 (322)^d^	3210 (537)^f^	.73

Abbreviations: App, application; PSS, Perceived Stress Scale.

^a^ n missing = 12.

^b^ n missing = 7.

^c^ n missing = 28.

^d^ n missing = 3.

^e^ n missing = 9.

^f^ n missing = 13.

* Significant values are in bold.

### App Usage and Pregnancy Outcomes

Table 3 provides the birthweight and gestational age at birth for both the app user cohort and the historical control. Birthweight and gestational age were similar for both app users and the historical control.

## DISCUSSION

This pilot study tested the feasibility and effectiveness of using a mindfulness app to reduce stress in pregnant patients. The study involved recruiting pregnant patients and asking them to maintain an app‐based mindfulness practice as well as complete perceived stress self‐assessment surveys. By comparing app users with a historical control cohort of pregnant patients who did not use the mindfulness app, we were able to gain insight about the effectiveness of this type of intervention on reducing stress. The high rate of recruitment of participants approached (84%) suggests that pregnant patients are eager for a nonpharmacologic intervention to reduce stress. Although not all enrolled patients committed to using the app, those patients who used the app for any amount of time tended to have reduced stress levels. Together, the results of this study demonstrated that mindfulness apps may be an effective mental health intervention for reducing perceived stress in pregnant patients. Given the limited adherence with both app usage and survey completion, there remains a question about feasibility. As this is a noninvasive, low‐cost treatment for mood disorders with seemingly no risk to patients, lower adherence is somewhat more acceptable when compared with other interventions. Still, future studies should address solutions to increase adherence and thus bolster feasibility of a mindfulness app‐based treatment.

Mindfulness‐based cognitive behavioral therapy is emerging as an effective treatment for stress in pregnant patients.^21^, ^22^ Recent studies suggest that this therapy does not need to be given in a traditional setting to be an effective treatment against stress and depression.^23^, ^28^, ^30^ However, the feasibility and effectiveness of a mindfulness app as a tool to treat stress in pregnant patients has been only minimally investigated to date. Our results indicate that an app‐based mindfulness practice may reduce stress in pregnant patients. Because apps are widely available and relatively inexpensive in comparison with individualized cognitive behavioral therapy, they can help bridge the gap in mental health access experienced in rural and impoverished communities.^18^ Furthermore, the coronavirus disease 2019 (COVID‐19) pandemic has increased the need for virtual mental health resources, particularly for at‐risk populations like pregnant patients.

This pilot study was limited by its small sample size and lack of randomized control group. All patients who enrolled in the study joined the app user group and were compared with a historical control cohort that was not given the same choice; thus, the app user cohort may have selected for patients who were highly motivated to reduce their stress levels. Future studies with expanded enrollment should feature an app user and control group that are each randomly assigned to prevent selection bias.

A major strength of this study was the ease of recruitment, with a high proportion of approached participants electing to enroll. Enrolled patients were instructed to use the app daily. Unfortunately, less than half of participants completed a single meditation through the app's platform; similarly, there was limited adherence with completion of follow‐up surveys. Of those patients who completed at least one meditation, the degree of app usage varied broadly from just a few minutes to several hours of cumulative use. Of 59 patients consented for the study, only 12 patients used the app and finished the follow‐up surveys. Limited adherence of enrolled patients is a consistent issue plaguing app‐based mental health intervention studies.^30^, ^31^ Interestingly, study participants who did not use the app had significantly higher gravidity and parity; it is certainly possible that increased childcare demands limited ability to consistently use the app for meditations. Future studies may improve adherence with both app usage and completion of follow‐up assessments by incentivizing these tasks. Despite variable adherence, those patients who used the app did have reduced PSS at their follow‐up assessments.

The results of this pilot study provide justification for pursuing further research into the effectiveness of an app‐based mental health practice in reducing stress for pregnant patients. This study assessed changes in perceived stress score as the primary outcome and gestational age at birth and birthweight as the secondary outcome. Future studies could incorporate objective stress measures (ie, cortisol) in addition to perceived stress measures (ie, PSS), and expand upon postbirth and neonatal follow‐up.

The COVID‐19 pandemic has increased the need for virtual mental health interventions that are easily accessed and affordable.^32^ Our study provides pilot evidence that mindfulness apps can be part of the arsenal of virtual tools that health care workers might use to encourage their pregnant patients when in need of stress reduction.

## CONCLUSION

Our study has added pilot data to an emerging field of virtual mindfulness interventions in pregnancy. Similar to other mindfulness research, we were limited by adherence; however, our study shows promising results in feasibility and desirability to patients. Although our sample size was small in this pilot work, we found that in a subset of motivated patients, an app‐based mindfulness practice significantly reduced perceived stress between the second and third trimesters. Prenatal mindfulness apps represent an important low‐intervention, low‐cost, highly accessible tool for managing perinatal mood and stress.

## CONFLICT OF INTEREST

Expectful provided free use of its mobile application for the purposes of this study. Otherwise, the authors have no conflicts of interest to disclose.
